# Differences in responses of grass carp to different types of grass carp reovirus (GCRV) and the mechanism of hemorrhage revealed by transcriptome sequencing

**DOI:** 10.1186/s12864-017-3824-1

**Published:** 2017-06-08

**Authors:** Libo He, Aidi Zhang, Yongyan Pei, Pengfei Chu, Yongming Li, Rong Huang, Lanjie Liao, Zuoyan Zhu, Yaping Wang

**Affiliations:** 1 State Key Laboratory of Freshwater Ecology and Biotechnology, Institute of Hydrobiology, Chinese Academy of Sciences, Wuhan, 430072 China; 2 University of Chinese Academy of Sciences, Beijing, 100049 China

**Keywords:** Grass carp, Grass carp reovirus, Transcriptome sequencing, Immune response, Hemorrhage

## Abstract

**Background:**

Grass carp is an important farmed fish in China that is affected by serious disease, especially hemorrhagic disease caused by grass carp reovirus (GCRV). The mechanism underlying the hemorrhagic symptoms in infected fish remains to be elucidated. Although GCRV can be divided into three distinct subtypes, differences in the pathogenesis and host immune responses to the different subtypes are still unclear. The aim of this study was to provide a comprehensive insight into the grass carp response to different GCRV subtypes and to elucidate the mechanism underlying the hemorrhagic symptoms.

**Results:**

Following infection of grass carp, GCRV-I was associated with a long latent period and low mortality (42.5%), while GCRV-II was associated with a short latent period and high mortality (81.4%). The relative copy number of GCRV-I remained consistent or decreased slightly throughout the first 7 days post-infection, whereas a marked increase in GCRV-II high copy number was detected at 5 days post-infection. Transcriptome sequencing revealed 211 differentially expressed genes (DEGs) in Group I (66 up-regulated, 145 down-regulated) and 670 (386 up-regulated, 284 down-regulated) in Group II. Gene Ontology (GO) and Kyoto Encyclopedia of Genes and Genomes (KEGG) enrichment analysis showed significant enrichment in the terms or pathways involved in immune responses and correlating with blood or platelets. Most of the DEGs in Group I were also present in Group II, although the expression profiles differed, with most DEGs showing mild changes in Group I, while marked changes were observed in Group II, especially the interferon-related genes. Many of the genes involved in the complement pathway and coagulation cascades were significantly up-regulated at 7 days post-infection in Group II, suggesting activation of these pathways.

**Conclusion:**

GCRV-I is associated with low virulence and a long latent period prior to the induction of a mild host immune response, whereas GCRV-II is associated with high virulence, a short latent period and stimulates a strong and extensive host immune response. The complement and coagulation pathways are significantly activated at 7 days post-infection, leading to the endothelial cell and blood cell damage that result in hemorrhagic symptoms.

**Electronic supplementary material:**

The online version of this article (doi:10.1186/s12864-017-3824-1) contains supplementary material, which is available to authorized users.

## Background

The grass carp (*Ctenopharyngodon idellus*) has been an important aquaculture species in China for over 60 years, accounting for more than 18% of total freshwater aquaculture production. The production of grass carp reached 5.5 million tons in 2014, making it the most highly consumed freshwater fish worldwide [1].

Grass carp hemorrhagic disease, caused by the grass carp reovirus (GCRV), is one of the most serious of these diseases [2]. GCRV, which was first isolated in China, belonging to the genus *Aquareovirus* of the family *Reoviridae* [3]. GCRV infects not only grass carp, but also rare minnow (*Gobiocypris rarus*), black carp (*Mylopharyngodon piceus*), and topmouth gudgeon (*Pseudorasbora parva*), causing hemorrhagic symptoms and death. Disease caused by GCRV outbreaks are frequent and result in huge economic losses in the aquaculture industry. Consequently, GCRV is of particular interest to fish breeding geneticists aiming to identify strategies for disease-resistant breeding [4–9].

Recently, the genome sequences of a number of GCRV strains isolated in China have been determined [10–12]. Sequence comparisons and analysis showed that GCRV could be divided into three distinct subtypes. GCRV-873, which is a representative strain of type I (GCRV-I), infected *C. idellus* kidney (CIK) cells and induced obvious cytopathic effects (CPE). GCRV-HZ08, which is a representative strain of type II GCRV (GCRV-II), resulted in 80% mortality in yearling fish, while no obvious CPEs were observed in GCRV-II infected CIK cells. Type III GCRV (GCRV-III) is not widely distributed in China and only one strain was found (GCRV-104), which also induced CPE in CIK cells.

Of the three types of GCRV, GCRV-873 was the first fish virus to be characterized and sequenced [13] and was used as the target in early studies focusing on disease-resistant breeding and virus-host interactions. However, recent studies showed that most of the GCRV isolated in Southern China are type II GCRVs, such as GCRV-HZ08, GCRV-GD108 and GCReV-109 [10–12]. GCRV types I and II both cause hemorrhagic disease in grass carp, although the hemorrhage mechanism is unknown. Moreover, these virus types have significantly different nucleotide sequences, viral-encoded protein structures, and pathogenicity in grass carp and CIK cells. Although many studies on GCRV have been conducted, most were restricted to investigation of the virus itself and differences in pathogenesis and immune responses to different types of GCRV in grass carp remain to be elucidated.

In this study, grass carp were infected with two types of GCRV (GCRV-I and GCRV-II) and the pathogenesis was investigated by transcriptome sequencing, real time quantitative PCR (RT-qPCR) and mortality rates. Details of the transcriptional events following GCRV infection have been reported previously [14]; therefore, the aim of the current study was to provide a comprehensive insight into the responses of grass carp to different types of GCRV and to reveal the mechanism underlying the hemorrhagic symptoms. Our study will provide guidance for the development of novel vaccines and disease-resistant breeding of grass carp.

## Methods

### Viruses

Two types of grass carp reovirus were used in the study. One GCRV strain, isolated in Honghu City, Hubei Province, China in May 2015, was classified as a type I GCRV due to the high similarity (97.3%) of the S2 segment to the typical type I strain, GCRV-873. Another GCRV strain, isolated in Huanggan city, also in Hubei Province, in July 2015, was classified as a type II GCRV due to the high similarity (98.4%) of the S2 segment to the typical type II strain, GCRV-HZ08. The two types of GCRV were designated GCRV-I and GCRV-II for the purposes of this study and both were diluted to the same titer (2.97 × 10^3^ RNA copy/μl) for use in experiments.

### Experimental fish

Healthy full-sib grass carp were used in the study at 3 months of age, weighing 3–5 g and with an average length of 8 cm. The fish were obtained from the Guan Qiao Experimental Station, Institute of Hydrobiology, Chinese Academy of Sciences, and acclimatized in aerated fresh water at 26–28 °C for one week before processing. Fish were fed with a commercial diet twice a day and water was exchanged daily. If no abnormal symptoms were observed, grass carp were selected for further study. Fish were then divided into three groups (approximately 150 per group) that were maintained in separate tanks.

### Virus challenge experiment and sample collection

After no abnormal symptoms were observed in the three groups, virus challenge experiments were carried out. Fish in the Groups I and II were infected with 200 μl GCRV-I (2.97 × 10^3^ RNA copy/μl) or 200 μl GCRV-II (2.97 × 10^3^ RNA copy/μl) by intraperitoneal injection, respectively, while fish from Group III were injected with 200 μl PBS as a Control group. At 1, 3, 5, and 7 days post-injection, 15 fish that contained three biological duplicates (*n* = 5 for each biological duplicate) from each group were collected and the kidneys were removed for analysis. The samples were designated I-1, I-3, I-5, I-7, II-1, II-3, II-5, II-7, c-1, c-3, c-5, and c-7 (three biological duplicates for each sample). The remaining fish were monitored carefully and the number of dead fish in each group was counted every day. The experiment was concluded and the total mortality was calculated when no mortality was recorded for seven consecutive days.

### RNA isolation, library construction and sequencing

RNA of kidneys that collected above was isolated using TRizol reagent (Invitrogen, USA) according the manufacturer’s protocol. RNA concentration was measured using the Qubit RNA assay kit (Life Technologies, USA), and integrity was assessed with the RNA Nano 6000 assay kit (Agilent Technologies, USA). RNA of sufficiently high quality was used in library construction. Sequencing libraries were generated using the NEBNext Ultra RNA library prep kit for Illumina (New England Biolabs, USA) following the manufacturer’s protocol. Briefly, mRNA was purified from total RNA using poly-T oligo-attached magnetic beads and fragmented by the NEBNext first strand synthesis reaction buffer (New England Biolabs). First strand cDNA was synthesized using a random hexamer primer and M-MuLV reverse transcriptase. Second strand cDNA synthesis was subsequently performed using DNA polymerase I and RNase H. After adenylation of the 3′ end of DNA fragments, NEBNext adaptors with a hairpin loop structure were ligated in preparation for hybridization. Subsequently, 3 μL USER enzyme (New England Biolabs, USA) was used with size-selected, adaptor-ligated cDNA at 37 °C for 15 min followed by 5 min at 95 °C prior to PCR using Phusion High-fidelity DNA polymerase, universal PCR primers and index (X) primer. Finally, PCR products were purified using an AMPure XP system and library quality was assessed using an Agilent Bioanalyzer 2100 system. Libraries were sequenced on an Illumina Hiseq X Ten platform and 150 bp pair-end reads were generated.

### Data analysis

Raw data reads in fastq format were initially processed using in-house perl scripts. In this step, clean data (clean reads) were obtained by removing adapter, poly-N and poor quality data. The Q20, Q30, and GC contents of the clean data were calculated, and all downstream analysis was performed using the clean high quality data.

Clean data were mapped to the grass carp reference genome [15] using TopHat2 software [16]. Allowing for two base mismatches in the mapping process, total mapped reads were calculated, and the mapped regions (exon, intron, and intergenic) were counted.

HTSeq software was used to count the number of reads mapped to each gene [17] and the reads per kilobase of the exon model per million mapped reads (RPKM) were calculated for each gene based on the length of the gene and the number of reads mapped to the gene [18].

### Differential expression analysis

Differential expression analysis of two groups/conditions was performed using the DESeq package [19]. The resulting *P*-values were adjusted using the Benjamini and Hochberg approach for controlling the false discovery rate. Genes with an adjusted *P*-value <0.05 (q value <0.05) in DESeq analysis were assigned as differentially expressed genes (DEGs).

Gene Ontology (GO) annotation of the genes was performed using ClueGO and CluePedia [20, 21]. In GO enrichment analysis, only categories with a low *P*-value (*P* < 0.05) were considered as enriched in the network as determined by two-sided hypergeometric statistical tests employing the Benjamini and Hochberg approach to false discovery rate correction.

The Kyoto Encyclopedia of Genes and Genomes (KEGG) database is used to provide high-level functional information on biological systems of molecules, cells, organisms and ecosystems, and is particularly powerful for the evaluation of large-scale molecular datasets generated by genome sequencing and other high-throughput experimental approaches [22]. In this study, KOBAS software was employed to test the statistical enrichment of DEGs in KEGG pathways [23]. KEGG terms with corrected *P <* 0.05 were considered to indicate statistical significance.

### Validation of DEGs by RT-qPCR

To confirm the reliability of data obtained by RNA-seq, 10 DEGs were selected for validation by RT-qPCR. The primers are listed in Additional file 1. Only primers with efficiency of 90%–110% were used for RT-qPCR analysis. First strand cDNAs were obtained using a random hexamer primer and the ReverTra Ace kit (Toyobo, Japan). RT-qPCR was carried out using a fluorescence quantitative PCR instrument (Bio-Rad, USA). Each RT-qPCR mixture contained 0.8 μL forward and reverse primers (for each primer), 1 μL template, 10 μL 2× SYBRgreen master mix (TOYOBO, Japan), and 7.4 μL ddH_2_O. Three replicates were included for each sample and the *β-actin* gene was used as an internal control to for normalization of gene expression. The program for RT-qPCR was as follows: 95 °C for 10 s, 40 cycles of 95 °C for 15 s, 55 °C for 15 s, and 72 °C for 30 s. Relative expression levels were calculated using the 2^-△△Ct^ method [24]. Data represent the mean ± standard deviation of three replicates.

### Statistical analysis

The statistical significance between Group I and Group II was determined by one-way ANOVA. Differences were considered significant at *P* < 0.05. *P* < 0.05 was denoted by *.

## Results

### Mortality of grass carp infected with GCRV-I or GCRV-II

**Fig. 1:**
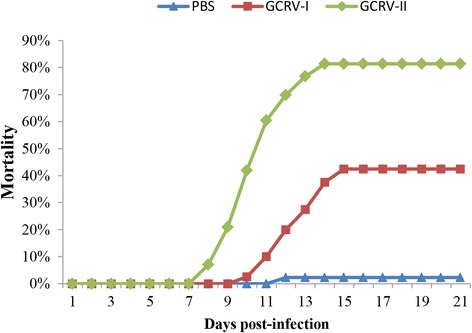
Cumulative mortality of fish. Fish were infected with 200 μl GCRV-I, GCRV-II, or PBS by intraperitoneal injection, respectively. The number of dead fish in each group was counted every day until no deaths were recorded for seven consecutive days; the total mortality was then calculated

**Fig. 2:**
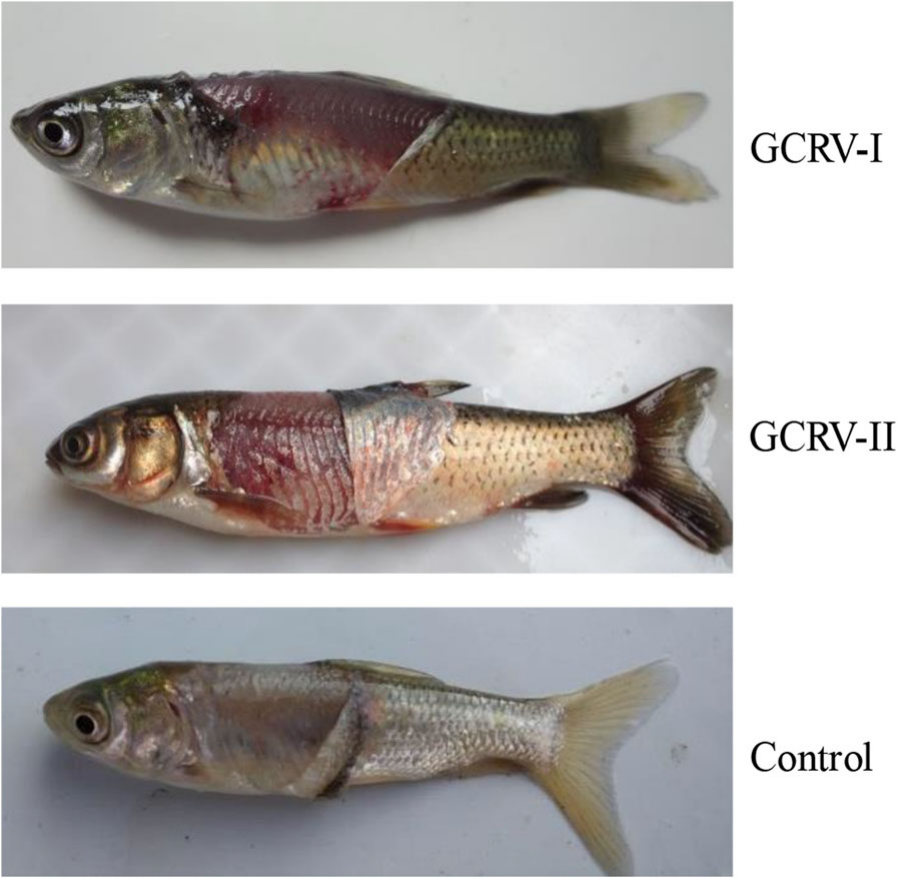
The hemorrhagic symptoms induced by GCRV. Images showing representative fish with typical muscular hemorrhagic symptoms in Groups I and II and the Control

The mortality curves of the three groups are shown in Fig. 1. In the GCRV-I-infected group, the total mortality of 42.5% was reached at 15 days post-infection, with the first fish dying at 10 days post-infection. However, in the GCRV-II-infected group, the total mortality of 81.4% was reached at 15 days, with the first death recorded as early as 8 days post-infection. In the Control group, two dead individuals were observed, giving a total mortality of 2.2%. Moreover, fish that died after infection with GCRV-I or GCRV-II showed hemorrhagic symptoms, especially in the muscle, whereas no hemorrhagic symptoms were observed in the Control group fish (Fig. 2).

### Relative copy numbers of the two types of GCRV in grass carp

**Fig. 3:**
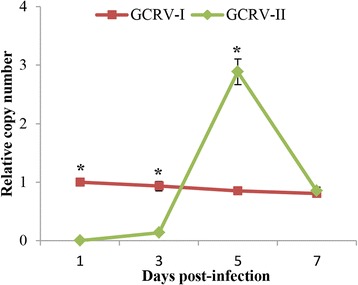
Relative copy number of GCRV in Group I and Group II. The relative copy numbers of GCRV in Groups I and II were examined by using specific primers for the S6 segment. The copy number of GCRV at 1 day post-infection in Group I was used as a reference for normalization. Data represent mean ± standard deviation of three replicates. Significant difference (*P* < 0.05) between the two groups was indicated with asterisks (*)

To determine dynamic changes in the levels of the two types of GCRV in infected fish, the relative copy numbers of the viruses were examined by RT-qPCR using specific primers for the S6 segments of the two types of GCRV. For convenience, the relative copy number of GCRV in one day post-infection in Group I was used as a reference for normalization. As shown in Fig. 3, the relative copy number of GCRV-I in Group I was consistent or decreased slightly throughout the experiment. In contrast, the relative copy number of GCRV-II in Group II was extremely low at 1 and 3 days post-infection, followed by a marked increase at 5 days post-stimulation and a reduction to levels similar to those of GCRV-I in Group I at 7 days post-infection. Obviously, the two types of GCRV showed different dynamic curves during infected fish.

### Preliminary analysis of transcriptome sequencing data

To further reveal the mechanism underlying the differences in sensitivity of grass carp to the two types of GCRV, we performed RNA-seq analysis on samples collected from three groups at different time-points post-infection. Three duplicates of each sample were processed, yielding a total of 36 libraries, which were sequenced on an Illumina Hiseq X Ten platform to generate 150 bp pair-end reads. As shown in Table 1, the raw reads, clean reads, clean base Q20, Q30, and mapped percent for each library were recorded (Table 1). All libraries gave Q20 ≥ 95%, Q30 ≥ 87%, and mapped percent ≥87%. These results confirmed the high quality of the sequencing data and suitability for further analysis. The sequencing data in this study have been deposited in the Sequence Read Archive (SRA) at the National Center for Biotechnology Information (NCBI) (accession number: SRP095827).

**Table 1:** Summary of sequencing data in the study

Sample name	Duplicates	Raw reads	Clean reads	Clean base (Gb)	Q20 (%)	Q30 (%)	Mapped (%)
I-1	a	43,959,191	42,775,222	6.4	96.48	89.53	88.26
	b	46,024,032	44,762,682	6.7	96.26	89.13	90.09
	c	42,180,866	41,017,933	6.2	96.44	89.39	90.27
I-3	a	43,175,970	42,015,916	6.3	96.22	88.98	90.80
	b	45,504,613	44,285,127	6.6	96.27	89.02	88.60
	c	44,279,976	43,069,779	6.5	96.21	88.92	89.89
I-5	a	42,440,949	41,273,811	6.2	96.23	89.1	90.55
	b	42,743,916	41,555,617	6.2	96.24	89.15	91.30
	c	44,936,847	43,704,884	6.6	96.18	89.03	88.96
I-7	a	42,302,608	41,142,107	6.2	96.32	89.28	89.19
	b	44,763,427	43,571,261	6.5	96.3	89.19	89.72
	c	43,916,414	42,749,930	6.4	96.34	89.29	91.32
II-1	a	45,999,850	44,785,102	6.7	96.22	89.01	87.18
	b	42,941,167	41,751,223	6.3	96.15	88.90	89.59
	c	44,268,235	43,109,075	6.5	96.38	89.33	89.47
II-3	a	46,881,460	45,686,432	6.9	96.37	89.37	91.09
	b	43,359,534	42,206,217	6.3	96.33	89.25	87.24
	c	42,641,431	41,496,401	6.2	96.37	89.37	89.72
II-5	a	43,358,527	42,183,431	6.4	96.28	89.16	89.88
	b	43,539,923	42,358,196	6.4	96.27	89.11	90.86
	c	44,335,544	43,109,720	6.5	96.13	88.86	87.38
II-7	a	45,463,769	44,240,714	6.6	96.23	89.2	89.75
	b	44,831,490	43,616,013	6.5	96.25	89.2	89.83
	c	43,771,968	42,577,304	6.4	96.24	89.2	90.88
C-1	a	43,428,497	42,233,801	6.3	96.13	88.9	87.67
	b	45,897,450	44,670,684	6.7	96.19	89.02	89.35
	c	43,842,260	42,652,596	6.4	96.32	89.27	89.79
C-3	a	45,354,222	44,137,729	6.6	96.02	88.58	90.71
	b	42,743,245	41,573,966	6.2	96.22	89.07	87.26
	c	44,613,776	43,405,844	6.5	96.13	88.83	89.09
C-5	a	44,397,927	43,195,411	6.5	96.21	89.14	89.99
	b	43,318,566	42,125,322	6.3	96.32	89.42	91.24
	c	45,299,655	44,111,640	6.6	96.28	89.2	88.44
C-7	a	44,182,777	42,985,305	6.4	95.72	87.96	89.51
	b	43,638,964	42,449,707	6.4	95.79	88.08	89.98
	c	45,255,896	44,734,541	6.7	95.6	87.67	91.07

### Identification of DEGs

**Fig. 4:**
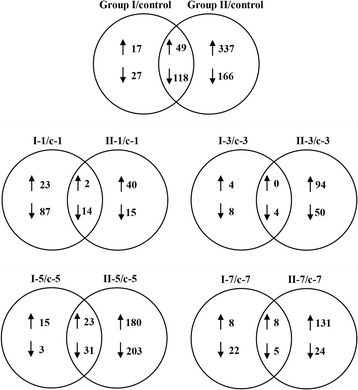
Venn diagram of differentially expressed genes between the two groups. Venn diagram showing the differentially expressed genes common to Groups I and II in total and at each time-point

DEGs among these samples were identified by subjecting the data to a series of paired-comparisons. In the analysis, samples from Group I (I-1, I-3, I-5, and II-7) and Group II (II-1, II-3, II-5, and II-7) were compared with samples that from the Control group at the corresponding time-points the same time. The numbers of DEGs identified from the different paired-comparisons are listed in Table 2. Comparisons with the Control group revealed 66 up-regulated and 145 down-regulated genes in Group I, whereas 386 up-regulated and 284 down-regulated genes were identified in Group II. Venn diagram analysis of the 49 up-regulated and 115 down-regulated genes found in both groups is shown in Fig. 4. In detail, 25, 4, 38 and 16 genes were un-regulated, whereas 102, 13, 35, and 28 genes were down-regulated in Group I at 1, 3 5 and 7 days post-infection, respectively. In Group II, 42, 94, 203, and 139 genes were up-regulated and 29, 54, 234, and 29 genes were down-regulated at 1, 3 5 and 7 days post-infection, respectively. Detailed information of these DEGs is shown in Additional file 2. The DEGs that could not be functionally annotated are listed as “unknown”.

**Table 2:** Summary of DEGs in different comparison

Comparisons	Details	Up	Down	Total
Group I/Control	I-1/c-1	25	101	126
	I-3/c-3	4	12	16
	I-5/c-5	38	34	72
	I/7-c-7	16	27	43
	In total	66	145	211
Group II/Control	II-1/c-1	42	29	71
	II-3/c-3	94	54	148
	II-5/c-5	203	234	437
	II/7-c-7	139	29	168
	In total	386	284	670

### GO and KEGG enrichment analysis of the DEGs

GO enrichment analysis was performed to investigate the possible roles of these DEGs. Of the three main categories (biological process, molecular function, and cellular component), most of the GO terms belonged to the biological process category, suggesting the occurrence of a series of molecular events in grass carp after GCRV infection. Many of the significantly enriched GO terms in both groups were involved in immune responses, such as defense response, response to organic substance, inflammatory response, acute inflammatory response, and innate immune response, indicating that grass carp respond strongly to both types of GCRV infection. Moreover, some GO terms associated with blood or platelets, such as blood microparticles, blood coagulation, platelet activation, and platelet degranulation, were also significantly enriched. The top five enriched GO terms in both groups are listed in Table 3 and details of the GO terms are shown in Additional file 3.

**Table 3:** Top five enriched GO terms and KEGG pathways of the differentially expressed genes in both groups

GO terms		
Groups	GO term	Corrected *P*-value
Group I	Extracellular space	2.85E-05
	Blood microparticle	6.39E-05
	Acute inflammatory response	1.01E-04
	Inflammatory response	2.10E-04
	Regulation of protein activation cascade	2.37E-04
Group II	Extracellular space	3.50E-19
	Blood microparticle	3.08E-13
	Extracellular organelle	3.87E-13
	Extracellular membrane-bound organelle	5.16E-13
	Extracellular vesicular exosome	5.56E-12
KEGG pathways		
Groups	KEGG pathways	Corrected *P*-value
Group I	Complement and coagulation cascades	2.09E-14
	*Staphylococcus aureus* infection	2.35E-04
	Glycolysis/gluconeogenesis	4.57E-04
	Vitamin digestion and absorption	0.001947
	Fat digestion and absorption	0.002017
Group II	Complement and coagulation cascades	1.26E-21
	*Staphylococcus aureus* infection	1.13E-06
	Glycolysis/gluconeogenesis	2.86E-04
	peroxisome proliferator-activated receptor (PPAR) signaling pathway	4.36E-04
	Graft-versus-host disease	9.54E-04

KEGG enrichment analysis was also performed for the DEGs in both groups. In general, many the significantly enriched KEGG terms were also involved in immune responses, such as complement and coagulation cascades, *Staphylococcus aureus* infection, the peroxisome proliferator-activated receptor (PPAR) signaling pathway, graft-versus-host disease, and allograft rejection. In addition, some terms involved in metabolism and biosynthesis were also significantly enriched. These terms included glycolysis/gluconeogenesis, phenylalanine, tyrosine and tryptophan biosynthesis, as well as vitamin digestion and absorption, and fat digestion and absorption. The top five enriched KEGG terms in both groups are also listed in Table 3 and details of the KEGG terms are shown in Additional file 4.

### Expression patterns of DEGs in the key GO terms

**Fig. 5:**
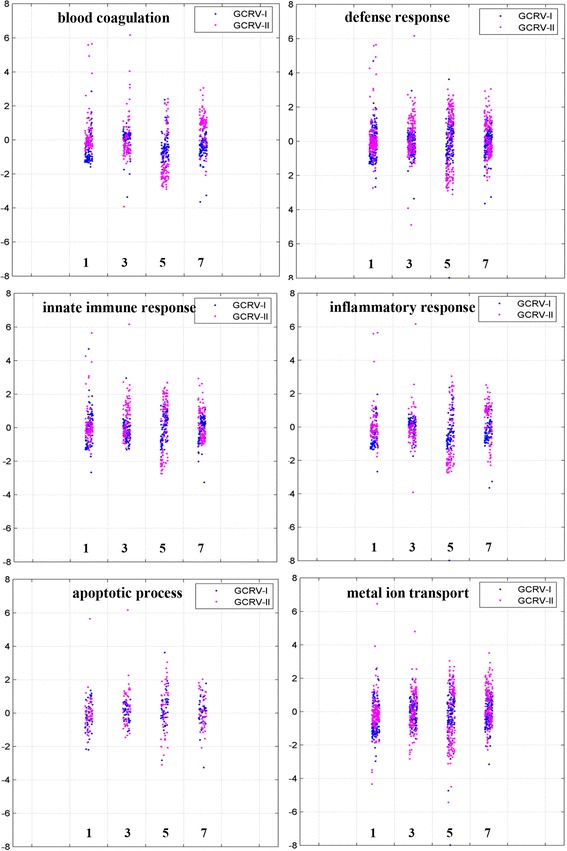
Scatterplots of gene expression pattern of differentially expressed genes (DEGs) in the selected GO terms. Scatterplots showing the log_2_fold changes in DEGs in both groups and comparison of the GO terms involved in blood coagulation, defense response, innate immune response, inflammatory response, apoptotic process, and metal ion transport. The X-axes means the days post-infection and the Y-axes indicates the values of log_2_fold change

The GO terms involved in blood coagulation, defense response, innate immune response, inflammatory response, apoptotic process, and metal ion transport were selected for further investigation. Comparisons at each time-point after infection revealed significant differences in the expression patterns of these DEGs in the two groups (Fig. 5). The DEGs in Group I showed mild change in expression at the four time-points, with log_2_fold changes ≤2 for most of genes. However, a significant variation in the expression levels of the DEGs in Group II was observed, with the most marked variation at 5 days post-infection. Many of DEGs in Group II showed log_2_fold changes ≥2 or even ≥4.

### Identification of DEGs shared between the two groups

Venn diagram analysis showed that the two groups shared 49 up-regulated and 118 down-regulated DEGs, regardless of the time-points post-infection. These shared DEGs were subjected to further analysis to investigate the common events after infection with different types of GCRV. Of the 49 up-regulated DEGs, many were involved in anti-viral immune response, blood coagulation, response to stress, protein degradation, antigen presentation, and transcription/translation. Although these DEGs were up-regulated in both groups, the expression patterns differed. The representative up-regulated DEGs are listed in Table 4. Of the down-regulated DEGs, many were involved in in blood coagulation, cytoskeleton, complement activation, iron transport, inflammatory response, and metabolism. The representative up-regulated DEGs are also listed in Table 4. Interestingly, the representative DEGs (both up-regulated and down-regulated) belonging to the same categories showed similar expression patterns in both groups. Details of these DEGs in both groups are shown in Additional file 5.

**Table 4:** Representative differentially expressed genes in both groups

Category	Gene name	Log2 fold change							
		Group I				Group II			
		1	3	5	7	1	3	5	7
Representative up-regulated genes in both groups									
Anti-viral immune response	C-C motif chemokine 7	1.1	−1.0	0.7	0.3	0.6	1.4	2.4	−1.5
	Interferon-induced with tetratricopeptide repeats 5	1.5	−0.3	1.2	0.3	0.3	2.4	2.4	-0.9
	C-X-C motif chemokine 11	0.6	−0.5	1.5	−0.2	−0.6	0.7	2.2	−1.7
Blood coagulation	Urokinase plasminogen activator surface receptor	1.3	−0.5	0.7	0.5	−0.5	3.3	2.2	-1.1
	CD9 antigen	0.3	−0.5	1.3	0.8	−0.1	1.1	2.1	0.1
Response to stress	Mitomycin radical oxidase	2.2	−1.3	1.4	−0.1	0.5	1.4	2.3	−0.8
	Hypoxia-inducible factor 3-alpha	−0.4	0.4	0.9	1.1	−0.1	0.4	0.7	1.4
	DNA damage-inducible transcript 4	−0.5	0.4	3.6	1.4	0.6	−0.4	1.7	2.0
Protein degradation	Polyubiquitin contains	1.4	−0.3	1.6	0.4	−0.1	2.3	2.7	−0.7
	Probable E3 ubiquitin-ligase HERC3	0.9	-0.7	1.2	0.8	0.0	1.8	1.9	−0.4
	Suppressor of cytokine signaling	0.9	0.1	1.0	0.2	−0.1	1.1	2.2	−0.5
Antigen presentation	H-2 class II histocompatibility E-S beta chain	1.5	−0.4	1.3	0.1	3.1	0.0	1.0	0.2
	H-2 class I histocompatibility Q8 alpha chain	4.7	3.0	0.2	0.7	3.0	0.6	2.1	−0.1
Translation/transcription	Threonine-tRNA ligase	1.5	−0.3	0.3	0.2	0.4	1.0	1.7	−0.3
	Transcriptional regulator Myc-1	0.3	0.3	1.3	0.9	−0.2	0.2	1.1	0.0
Enzyme activity	Helicase with zinc finger domain 2	1.0	−0.5	1.4	0.7	0.1	2.2	2.2	−0.9
	Tetraacyldisaccharide 4′-kinase	0.3	−0.4	1.1	0.2	−0.2	1.3	1.6	−0.7
Representative down-regulated genes in both groups									
Blood coagulation	Hemopexin	−1.3	1.0	−1.3	−1.6	0.6	0.3	−2.3	2.6
	APOA1, apolipo A-I	−0.4	-1.7	−1.9	−3.6	−0.6	−3.9	−2.7	−1.1
	Fibrinogen gamma chain	−1.2	0.3	−0.7	−0.6	−0.2	0.1	-1.8	1.9
Cytoskeleton	Myosin-8	−2.2	0.2	−2.8	−1.6	−0.4	−0.4	−3.1	0.6
	Actin-binding protein	−1.7	−0.5	−0.9	−1.0	−0.5	−2.3	−2.0	0.1
	Myoglobin	−0.7	0.9	−4.7	0.0	−0.8	1.6	-5.4	0.0
Complement activation	Complement C3 beta chain	−1.2	0.3	−1.0	−0.8	0.0	−0.6	−2.5	0.9
	Complement C1q 4	−0.9	−1.5	−2.4	−0.1	1.9	−1.8	−2.6	2.5
	Complement component C9	−1.0	0.0	−0.7	−0.9	1.0	−0.4	-1.9	1.2
Iron transport	Serotransferrin-1	−1.0	0.2	−1.6	−1.2	1.2	−0.7	−2.6	1.6
	Ferritin M	−0.7	-0.8	0.6	−1.0	−1.8	−2.0	−0.9	−0.1
	Hepcidin-1	−1.4	0.4	−0.3	−1.1	−0.4	0.2	-2.6	1.5
Inflammatory response	Alpha-2-HS-glyco	−1.2	0.3	−0.8	−0.5	−0.3	−0.6	−2.2	0.9
	Plasma protease C1 inhibitor	−1.1	0.1	−0.8	−0.6	0.2	−0.5	−1.5	0.9
	Endothelin B receptor	0.0	0.0	−8.0	0.0	9.7	0.0	-8.1	0.0
Metabolism	Fructose-bisphosphate aldolase B	−1.2	0.6	−0.5	−1.0	−0.6	−0.7	−2.0	−0.2
	Fructose-1,6-bisphosphatase 1	−1.2	0.6	−0.3	−0.8	−0.9	−0.8	−2.3	0.4
	Glyceraldehyde-3-phosphate dehydrogenase	−1.2	0.4	0.1	−0.6	−0.4	−1.2	−2.1	0.9

### DEGs at 1 day post-infection

Comparison of the DEGs in the two groups at corresponding time-points is difficult because of the differences in the course of infection of the two types of GCRV. We selected the DEGs at day 1 post-infection for comparison because this time-point represents the initial stage of infection. At this time-point, 25 up-regulated and 101 down-regulated DEGs were found in Group I, while 42 up-regulated and 29 down-regulated DEGs were identified in Group II. Despite the many differences in the up-regulated DEGs between the two groups, many were categorized as immune response, response to stress, antigen presentation, and blood coagulation. In addition, most of the DEGs in Group I showed mild upregulation, with log_2_ fold changes ≤2, whereas those in Group II were intensely up-regulated, with log_2_ fold changes ≥2. The DEGs with log_2_ fold changes ≥2 in Group I and ≥3 in Group II are shown in Table 5. There were fewer down-regulated DEGs in Group II than in Group I. Many of the DEGs were involved in iron transport, transcription/translation, cytoskeleton, enzyme activity, and binding activity, suggesting inhibition of the host transcription/ translation machinery by GCRV. Interestingly, many genes encoding the middle subunit of ferritin were significantly down-regulated. The DEGs with log_2_fold changes ≤ − 2 in both groups are shown in Table 5.

**Table 5:** Significantly differentially expressed genes in both groups at 1 day post-infection

	Groups	Gene name	Log2 fold change
Up-regulated	Group I	H-2 class I histocompatibility Q8 alpha chain	4.74
		Urokinase plasminogen activator surface receptor	2.90
		Tumor necrosis factor receptor superfamily member 11B	2.58
		GTP-binding A	2.34
		Mitomycin radical oxidase	2.27
		Unknown	2.14
	Group II	Endothelin B receptor	9.65
		Stonustoxin subunit alpha	7.63
		Unknown	6.46
		Natterin	5.87
		Ribonuclease-like 3 Short = RNase ZF-3	5.64
		Haptoglobin alpha chain	5.59
		Perforin-1	4.93
		CD59 glycoprotein	4.27
		Endothelial cell-specific molecule 1	4.07
		Serum amyloid A-5	3.92
		H-2 class II histocompatibility E-S beta chain	3.09
		Phloem protein 2-like A3	3.05
Down-regulated	Group I	Neoverrucotoxin subunit beta	−3.33
		Neoverrucotoxin subunit alpha	−2.96
		Arginase-1	−2.96
		Unknown	−2.67
		Coenzyme Q-binding COQ10 mitochondrial	−2.34
		Amine sulfotransferase	−2.26
		Unknown	−2.22
		Probable DNA polymerase partial (mitochondrion)	−2.20
		Myosin-8	−2.15
	Group II	GTPase IMAP family member 5	−5.10
		Ferritin, middle subunit	−4.33
		Ferritin, middle subunit	−3.63
		Ferritin, middle subunit	−3.49
		Olfactomedin-4	−2.18

### Expression patterns of key interferon-related genes

**Fig. 6:**
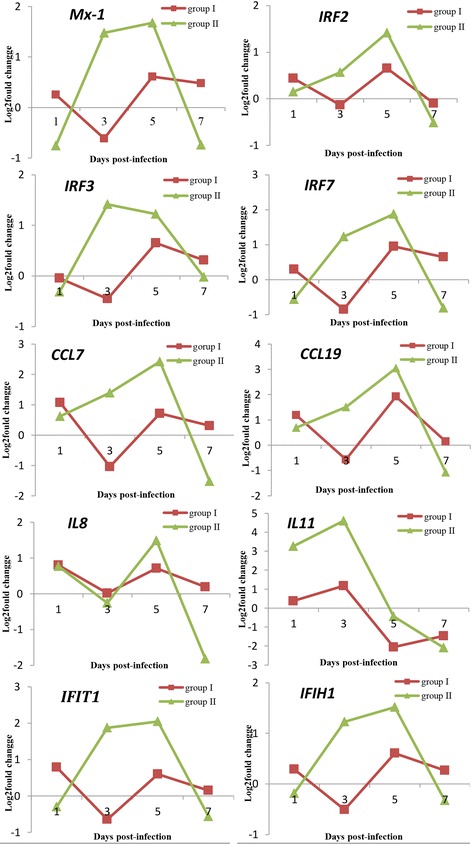
Expression patterns of key genes involved in immune responses. The expression patterns of immune genes *Mx-1*, *IRF2*, *IRF3*, *IRF7*, *CCL7*, *CCL19*, *IL8*, *IL11*, *IFIT1*, and *IFIH1* in both groups

The interferon system plays a critical role in both innate and adaptive immune responses to virus infection [25–27] and are particularly involved in fish responses to GCRV [28, 29]. Therefore, the expression patterns of key interferons and interferon-stimulated genes were examined in this study. These genes included interferon inducible Mx protein 1 (*Mx-1*), interferon regulatory factor 2 (*IRF2*), interferon regulatory factor 3 (*IRF3*), interferon regulatory factor 7 (*IRF7*), C-C motif chemokine 7 (*CCL7*), C-X-C motif chemokine 11 (*CXCL11*), interleukin 8 (*IL8*), interleukin 11 (*IL11*), interferon- induced protein with teratricopeptide repeats 1 (*IFIT1*), and interferon-induced helicase C domain-containing 1(*IFIH1*). As shown in Fig. 6, Groups I and II showed differences in the overall expression patterns of these genes. In Group I, most of genes showed log_2_fold changes ≤1 at all time-points post-infection, while more marked dynamic changes in expression of the genes was observed in Group II. Specifically, most of genes showed log_2_fold change ≥1 or even ≥2 at 3 or 5 days post-infection, whereas the expression level decreased at 7 days post-infection.

### DEGs involved in complement and coagulation cascades

**Fig. 7:**
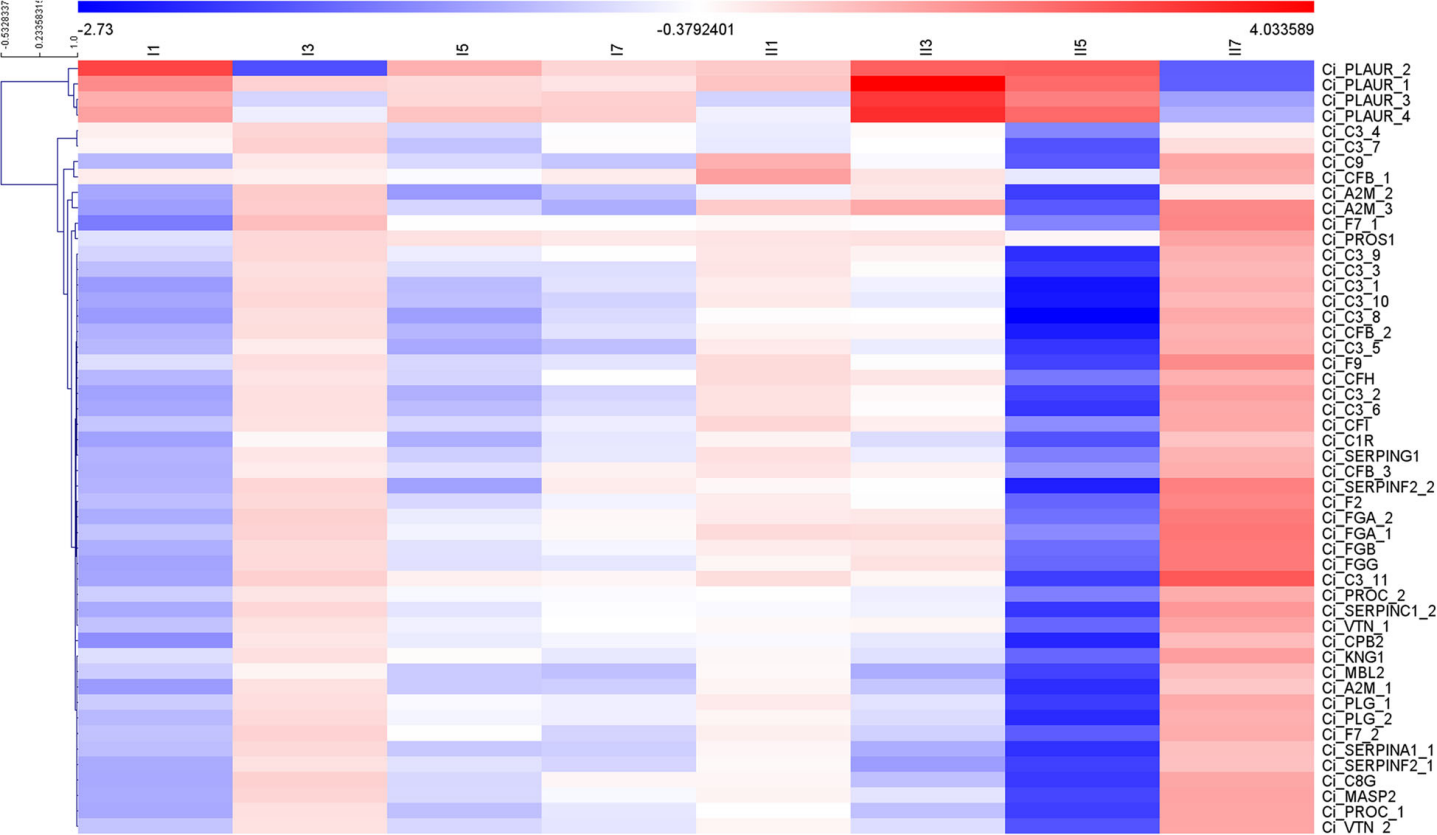
Heatmap of DEGs involved in the complement pathway and coagulation cascades. Heatmap showing log_2_fold changes in differentially expressed genes in both groups involved in the complement pathway and coagulation cascades

KEGG enrichment analysis showed that the “complement and coagulation cascades” pathway was the most significantly enriched in both groups. The expression patterns of the DEGs involved in these pathways were further investigated to elucidate the mechanism of hemorrhage in infected fish (Fig. 7). Interestingly, most of the DEGs showed similar expression patterns in each group, with the exception of the urokinase plasminogen activator surface receptor (*PLAUR*) gene, which showed an opposing expression pattern compared with other DEGs. The DEGs in Group I showed only slight changes in expression, with it increased levels detected at 3 days and decreased expression at 1, 5, and 7 days post-infection. Most of the DEGs in Group II showed mild changes at 1 and 3 days post-infection. However, significantly decreased expression of the DEGs was observed at 5 days post-infection, followed by marked upregulation at 7 days post-stimulation, suggesting activation of the “complement and coagulation cascades” pathways.

### Confirmation of DEGs by RT-qPCR

**Fig. 8:**
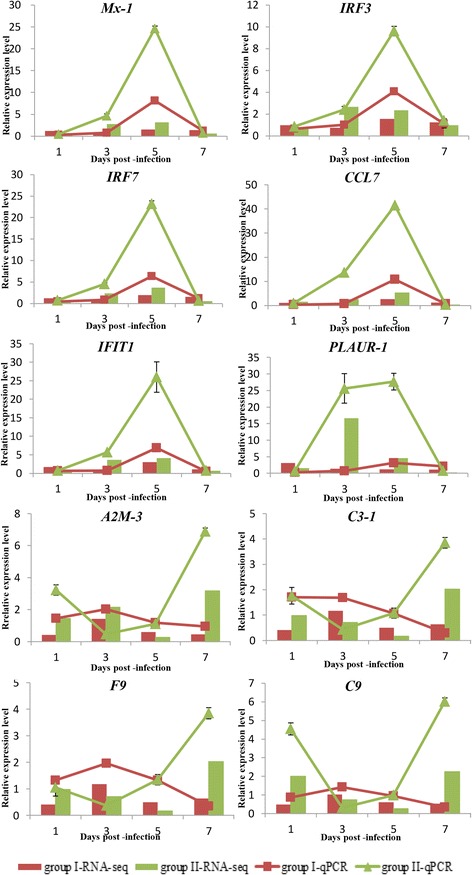
Confirmation of the transcriptome sequencing data by RT-qPCR. Five DEGs participating in immune responses and five DEGs involved in “complement and coagulation cascades” were selected for RT-qPCR. The relative expression levels of DEGs was calculated as the ratio of gene expression level relative to that in the Control group at the same time-point. All data represent the mean ± standard deviation of three replicates

To confirm the RNA-seq data, a total of 10 DEGs (5 involved in “immune response” and 5 involved in “complement and coagulation cascades”) were selected for RT-qPCR analysis. These genes included *Mx-1*, *IRF3*, *IRF7*, *CCL7*, *IFIT1*, *PLAUR-1,* alpha-2-macroglobulin 3 (*A2M-3*), complement C3c alpha chain fragment 1 (*C3–1*), coagulation factor IX (*F9*), and complement component 9 (*C9*). The relative expression levels of DEGs in Groups I and II were calculated as the ratio of gene expression levels relative to those in the Control group at the corresponding time-point. As shown in Fig. 8, overall, the expression patterns of all 10 DEGs identified by qPCR were similar to those obtained in RNA-seq analyses, although the relative expression levels were not completely consistent. Moreover, RT-qPCR also showed that the *PLAUR-1* gene expression pattern was opposite to that of the other genes that involved in “complement and coagulation cascades”. Therefore, the results of the RT-qPCR analysis confirmed the reliability and accuracy of the RNA-seq data.

## Discussion

In the study, we used RNA-seq to elucidate the the mechanism of hemorrhage and the different immune responses induced in grass carp infected with GCRV-I and GCRV-II.

Mortality analysis showed that grass carp infected with GCRV-I began to die at 10 days post-infection with a total mortality of 42.5%, whereas the first grass carp infected with GCRV-II died at 8 days post-infection and the total mortality was much higher, at 81.4%. The results suggested that the GCRV-II subgroup is associated with high virulence and a short latent period, while the GCRV-I subgroup is associated with low virulence and a longer latent period. Interestingly, changes in the relative copy numbers of the two types of GCRV during the course of infection showed results consistent with the theory mentioned in the above. The copy number of GCRV-I remained stable or even decreased slightly from day 1 to day 7 post-infection, whereas GCRV-II showed a rapid increase in copy number at 5 days post-stimulation. High virulence, short latent period, and high mortality could accelerate the spread of virus [30]. Our results explained that why the GCRV isolated recently in China belonged to GCRV-II rather than GCRV-I [10–12]. Moreover, the number of DEGs in the two groups is also consistent with the relative copy numbers. It can be speculated that fewer DEGs were identified in Group I due to the low copy numbers at the four time-points, while numerous DEGs were discovered in Group II, especially at 5 days post-infection due to the high GCRV-II copy number of at this time-point. The consistency of these data suggests the reliability of our experimental design.

Furthermore, the relatively low number of DEGs found in Group I compared with Group II suggest the induction of a strong and extensive response in Group II compared with the relatively mild response in Group I. The changes in DEGs in Group II were more dramatic than those in Group I. The variation in the expression pattern of DEGs among the selected GO terms was also greater in Group II than that in Group I. However, most of the DEGs in Group I were also identified in Group II, although the differences in expression patterns were consistent with the variation in the course of infection between the two groups. Therefore, we hypothesized that GCRV-I and GCRV-II induced similar responses in grass carp, although the response to GCRV-II was more robust and extensive. Previous studies revealed similar results in rainbow trout, which showed similar responses to high and low virulence strains of infectious hematopoietic necrosis virus (IHNV), although the level of the response and DEG profiles differed [31, 32].

Most of the up-regulated DEGs common to Groups I and II were associated with functions in anti-viral immune responses, blood coagulation, response to stress, protein degradation, antigen presentation, and transcription/translation, suggesting that a series of events, especially immune responses, occur after infection regardless of the GCRV subtype. Many of the down-regulated DEGs common to both Groups I and II were found to be involved in cytoskeleton, metabolism, iron transport, enzyme activity, and binding activity. This association was particularly marked in Group I at 1 day post-infection. The association of up-regulated DEGs with protein degradation, while down-regulated DEGs were associated with metabolism, enzyme activity, and binding activity suggests that the host translation machinery is hjjacked or shut-down by GCRV to facilitate the replication and spread of the virus. A similar phenomenon has been observed in other fish after virus infection [33]. Interestingly, DEGs involved in cytoskeleton and iron transport were down-regulated in both Groups I and II. These observations are consistent with reports that actin cytoskeletal dynamics are important for T lymphocyte activation and migration [34, 35] and the important role of iron homeostasis in the host response to infection [36–38].

GCRV causes hemorrhagic symptoms in infected fish, although the mechanism remains unknown. Interestingly, GO enrichment analysis revealed that some GO terms associated with blood or platelets, such as blood microparticles, blood coagulation, platelet activation, and platelet degranulation were significantly enriched, especially in Group II, indicating the existence of a correlation between these GO terms and the hemorrhagic symptoms. Moreover, KEGG analysis showed that DEGs associated with “complement and coagulation cascades” were the most significantly enriched in both groups. A heatmap of DEGs in this pathway showed only slight variation in log_2_fold changes in expression in Group I, whereas marked variation was observed in Group II. In addition, most of the DEGs in Group II were significantly up-regulated at 7 days post-infection, indicating that this pathway is highly activated. It has been reported that complement and coagulation systems play an important role in innate immunity [39, 40]; however, many studies have also shown that hyperactivity of the complement cascade can lead to endothelial and blood cell damage, resulting in platelet activation and aggregation, hemolysis, as well as prothrombotic and inflammatory changes [41, 42]. Thus, the significant activation of complement and coagulation cascades in Group II at 7 days post-infection may account for the hemorrhagic symptoms that appeared at 8 days post-infection in Group II.

## Conclusions

In conclusion, the results of this study provide an improved understanding of the differences in the pathogenesis and host immune response to GCRV-I and GCRV-II and the mechanism of hemorrhagic symptoms caused by these viruses. The GCRV-I subtype is associated with low virulence, a long latent period, and a mild host immune response, whereas the GCRV-II subtype is associated with relatively high virulence, a short latent period and a robust and extensive immune response. The significant activation of the pathway complement and coagulation pathways at 7 days post-infection, accounts for the damage to endothelial and blood cells that cause the hemorrhagic symptoms. This information forms the basis of further studies aimed at developing novel vaccines and determining strategies for disease-resistant breeding of grass carp.

## Additional files

Additional file 1: Sequences and efficiencies of primers used in RT-qPCR analysis. (DOCX 15 kb)

Additional file 2: Detailed information of the differentially expressed genes in the two groups. (RAR 360 kb)

Additional file 3: Detailed information of the GO terms of the differentially expressed genes in the two groups. (XLSX 1291 kb)

Additional file 4: Detailed information of the KEGG pathways of the differentially expressed genes in the two groups. (XLSX 42 kb)

Additional file 5: Detailed information of the common differentially expressed genes in the two groups. (XLSX 22 kb)

## Abbreviations

A2M-3: alpha-2-macroglobulin 3
C3–1: complement C3c alpha chain fragment 1
C9: complement component 9
CCL19: C-C motif chemokine 19
CCL7: C-C motif chemokine 7
CIK: *C. idellus* kidney
CPE: cytopathic effect
CXC11: C-X-C motif chemokine 11
DEGs: differentially expressed genes
F2: coagulation factor II
F9: coagulation factor IX
GCHV: grass carp hemorrhage virus
GCRV: grass carp reovirus
GO: Gene Ontology
IFIH1: and interferon-induced helicase C domain- containing 1
IFIT1: interferon- induced protein with tetratricopeptide repeats 1
IHNV: infectious hematopoietic necrosis virus
IL11: interleukin 11
IL8: interleukin 8
IRF2: interferon regulatory factor 2 (*IRF2*)
IRF3: interferon regulatory factor 3
IRF7: interferon regulatory factor 7
KEGG: Kyoto encyclopedia of genes and genomes
Mx-1: interferon inducible Mx protein 1
Mx-2: interferon inducible Mx protein 2
PLAUR: urokinase plasminogen activator surface receptor
RPKM: reads per kilobases per million reads
RT-qPCR: real time quantitative PCR

## References

[1] Food and Agriculture Organization of the United Nations. Fishery and Aquaculture Statistics Yearbook. Rome: Food and Agriculture Oranization of the United Nations; 2016.

[2] Rao Y, Su J. Insights into the antiviral immunity against grass carp (*Ctenopharyngodon idella*) reovirus (GCRV) in grass carp. J Immunol Res. 2015;670437. 10.1155/2015/670437 PMC4337036 25759845

[3] Attoui H, PPC M, Becnel J, Belaganahalli S, Bergoin M, Brussaard CP, King AMQ, Adams MJ, Carstens EB, Lefkowitz EJ (2012). Family Reoviridae. Virus Taxonomy: Ninth Report of the International Committee on Taxonomy of Viruses.

[4] Zeng W, Wang Y, Liang H, Liu C, Song X, Shi C (2014). A one-step duplex rRT-PCR assay for the simultaneous detection of grass carp reovirus genotypes I and II. J Virol Methods.

[5] Jian JC, Wang Y, Yan XY, Ding Y, Wu ZH, Lu YS (2013). Molecular cloning and prokaryotic expression of vp5 gene of grass carp reovirusstrain GCRV096. Virus Genes.

[6] Jing HL, Zhang LF, Fang ZZ, Xu LP, Zhang M, Wang N (2014). Detection of grass carp reovirus (GCRV) with monoclonal antibodies. Arch Virol.

[7] Zhou Y, Fan YD, Zeng LB, Ma J (2013). Prokaryotic expression and immunoassay of grass carp reovirus capsid VP6 protein. Acta Virol.

[8] Zhu B, Liu GL, Gong YX, Ling F, Wang GX (2015). Protective immunity of grass carp immunized with DNA vaccine encoding the vp7 gene of grass carp reovirus using carbon nanotubes as a carrier molecule. Fish Shellfish Immunol.

[9] Xu D, Song L, Wang H, Xu X, Wang T, Lu L (2015). Proteomic analysis of cellular protein expression profiles in response to grass carp reovirus infection. Fish Shellfish Immunol.

[10] Wang Q, Zeng W, Liu C, Zhang C, Wang Y, Shi C (2012). Complete genome sequence of a reovirus isolated from grass carp, indicating different genotypes of GCRV in China. J Virol.

[11] Fan Y, Rao S, Zeng L, Ma J, Zhou Y, Xu J (2013). Identification and genomic characterization of a novel fish reovirus, Hubei grass carp disease reovirus, isolated in 2009 in China. J Gen Virol.

[12] Pei C, Ke F, Chen ZY, Zhang QY (2014). Complete genome sequence and comparative analysis of grass carp reovirus strain 109 (GCReV-109) with other grass carp reovirus strains reveals no significant correlation with regional distribution. Arch Virol.

[13] Fang Q, Attoui H, Cantaloube JF, Biagini P, Zhu Z, de Micco P (2000). Sequence of genome segments 1, 2, and 3 of the grass carp reovirus (Genus Aquareovirus, family Reoviridae). Biochem Biophys Res Commun.

[14] Shi M, Huang R, Du F, Pei Y, Liao L, Zhu Z (2014). RNA-seq profiles from grass carp tissues after reovirus (GCRV) infection based on singular and modular enrichment analyses. Mol Immunol.

[15] Wang Y, Lu Y, Zhang Y, Ning Z, Li Y, Zhao Q (2015). The draft genome of the grass carp (*Ctenopharyngodon idellus*) provides insights into its evolution and vegetarian adaptation. Nat Genet.

[16] Kim D, Pertea G, Trapnell C, Pimentel H, Kelley R, Salzberg SL (2013). TopHat2: accurate alignment of transcriptomes in the presence of insertions, deletions andgene fusions. Genome Biol.

[17] Anders S, Pyl PT, Huber W. HTSeq-A Python framework to work with high-throughput sequencing data. bioRxiv. 2014; doi:10.1101/002824. 10.1093/bioinformatics/btu638 PMC4287950 25260700

[18] Mortazavi A, Williams BA, McCue K, Schaeffer L, Wold B (2008). Mapping and quantifying mammalian transcriptomes by RNA-Seq. Nat Methods.

[19] Anders S, Huber W (2010). Differential expression analysis for sequence count data. Genome Bio.

[20] Bindea G, Mlecnik B, Hackl H, Charoentong P, Tosolini M, Kirilovsky A (2009). Clue GO: a Cytoscape plug-in to decipher functionally grouped gene ontology and pathway annotation networks. Bioinformatics.

[21] Bindea G, Galon J, Mlecnik B (2013). CluePedia Cytoscape plugin: pathway insights using integrated experimental and in silico data. Bioinformatics.

[22] Kanehisa M, Araki M, Goto S, Hattori M, Hirakawa M, Itoh M (2008). KEGG for linking genomes to life and the environment. Nucleic Acids Res.

[23] Xie C, Mao X, Huang J, Ding Y, Wu J, Dong S (2011). KOBAS 2.0:web server for annotation and identification of enriched pathways and diseases. Nucleic Acids Res.

[24] Livak KJ, Schmittgen TD (2001). Analysis of relative gene expression data using real-time quantitative PCR and the 2(−Delta Delta C (T)) Method. Methods.

[25] Robertsen B (2006). The interferon system of teleost fish. Fish Shellfish Immunol.

[26] Poynter SJ, DeWitte-Orr SJ (2016). Fish interferon-stimulated genes: The antiviral effectors. Dev Comp Immunol.

[27] Langevin C, Aleksejeva E, Passoni G, Palha N, Levraud JP, Boudinot P (2013). The antiviral innate immune response in fish: evolution and conservation of the IFN system. J Mol Biol.

[28] Zhang YB, Jiang J, Chen YD, Zhu R, Shi Y, Zhang QY (2007). The innate immune response to grass carp hemorrhagic virus (GCHV) in cultured *Carassius auratus* blastulae (CAB) cells. Dev Comp Immunol.

[29] Yu FF, Zhang YB, Liu TK, Liu Y, Sun F, Jiang J (2010). Fish virus-induced interferon exerts antiviral function through Stat1 pathway. Mol Immunol.

[30] Kumberger P, Frey F, Schwarz US, Graw F (2016). Multiscale modeling of virus replication and spread. FEBS Lett.

[31] Peñaranda MM, Purcell MK, Kurath G (2009). Differential virulence mechanisms of infectious hematopoietic necrosis virus in rainbow trout (*Oncorhynchus mykiss*) include host entry and virus replication kinetics. J Gen Virol.

[32] Purcell MK, Marjara IS, Batts W, Kurath G, Hansen JD (2011). Transcriptome analysis of rainbow trout infected with high and low virulence strains of infectious hematopoietic necrosis virus. Fish Shellfish Immunol.

[33] Robledo D, Taggart JB, Ireland JH, McAndrew BJ, Starkey WG, Haley CS (2016). Gene expression comparison of resistant and susceptible Atlantic salmon fry challenged with Infectious Pancreatic Necrosis virus reveals a marked contrast in immune response. BMC Genomics.

[34] Samstag Y, Eibert SM, Klemke M, Wabnitz GH (2003). Actin cytoskeletal dynamics in T lymphocyte activation and migration. J Leukoc Biol.

[35] Pulgar R, Hödar C, Travisany D, Zuñiga A, Domínguez C, Maass A (2015). Transcriptional response of Atlantic salmon families to Piscirickettsia salmonis infection highlights the relevance of the iron-deprivation defence system. BMC Genomics.

[36] Doherty CP (2007). Host-pathogen interactions: the role of iron. J Nutr.

[37] Cherayil BJ (2011). The role of iron in the immune response to bacterial infection. Immunol Res.

[38] Sikorska K (2016). The iron homeostasis network and hepatitis C virus - a new challenge in the era of directly acting antivirals. Virulence.

[39] Foley JH (2016). Examining coagulation-complement crosstalk: complement activation and thrombosis. Thromb Res.

[40] Calame DG, Mueller-Ortiz SL, Wetsel RA (2016). Innate and adaptive immunologic functions of complement in the host response to Listeria monocytogenes infection. Immunobiology.

[41] Hamad OA, Bäck J, Nilsson PH, Nilsson B, Ekdahl KN (2012). Platelets, complement, and contact activation: partners in inflammation and thrombosis. Adv Exp Med Biol.

[42] Meri S (2013). Complement activation in diseases presenting with thrombotic microangiopathy. Eur J Intern Med.

