# Supplementary material for: Differences in responses of grass carp to different types of grass carp reovirus (GCRV) and the mechanism of hemorrhage revealed by transcriptome sequencing
Source: BMC Genomics. 2017 Jun 8;18:452. doi: 10.1186/s12864-017-3824-1 (PMC5465539; doi:10.1186/s12864-017-3824-1)
Supplement: Supplementary file 1 — Sequences and efficiencies of primers used in RT-qPCR analysis. (DOCX 15 kb) [file 12864_2017_3824_MOESM1_ESM.docx]

**Additional file 1 Primer sequences that used in the study**

| **Primers** | **Sequence (5’-3’)** | **efficiency** |
| --- | --- | --- |
| GCRV-I-F | CTCCTCCTTCACAACCACCAC | 101.5% |
| GCRV-I-R | AGCGAGCAAGCTCTTCCAGT |  |
| GCRV-II-F | AGCGCAGCAGGCAATTACTATCT | 99.6% |
| GCRV-II-R | ATCTGCTGGTAATGCGGAACG |  |
|  |  |  |
|  |  |  |
|  |  |  |
